# Transcriptional Regulation of Heart Development in Zebrafish

**DOI:** 10.3390/jcdd3020014

**Published:** 2016-04-09

**Authors:** Fei Lu, Adam D. Langenbacher, Jau-Nian Chen

**Affiliations:** Department of Molecular, Cell and Developmental Biology, University of California, Los Angeles, CA 90095, USA

**Keywords:** zebrafish, heart development, transcription factors, epigenetic regulators

## Abstract

Cardiac transcription factors orchestrate the complex cellular and molecular events required to produce a functioning heart. Misregulation of the cardiac transcription program leads to embryonic developmental defects and is associated with human congenital heart diseases. Recent studies have expanded our understanding of the regulation of cardiac gene expression at an additional layer, involving the coordination of epigenetic and transcriptional regulators. In this review, we highlight and discuss discoveries made possible by the genetic and embryological tools available in the zebrafish model organism, with a focus on the novel functions of cardiac transcription factors and epigenetic and transcriptional regulatory proteins during cardiogenesis.

## 1. Introduction

Cardiac morphogenesis and the maintenance of cardiac physiology require dynamic and carefully coordinated transcription programs. Studies in pluripotent stem cells and multiple model organisms have delineated critical signaling events involving Bmp, Fgf, Wnt, and Notch that induce the expression of a set of cardiogenic transcription factors to drive cardiac specification and differentiation (for review see [1,2,3]). Recent studies have revealed an additional layer of epigenetic regulation that contributes to the tight temporal and spatial control of cardiac gene expression during development (for review see [4,5,6]). Misregulation of cardiac transcription programs not only results in severe developmental defects, but is also associated with many congenital heart diseases (CHD), the leading inborn cause of mortality in infants. A deeper mechanistic understanding of the gene programs governing normal heart development will provide insights into pathogenic events leading to CHD and may result in the development of new therapeutic strategies.

**Figure 1:**
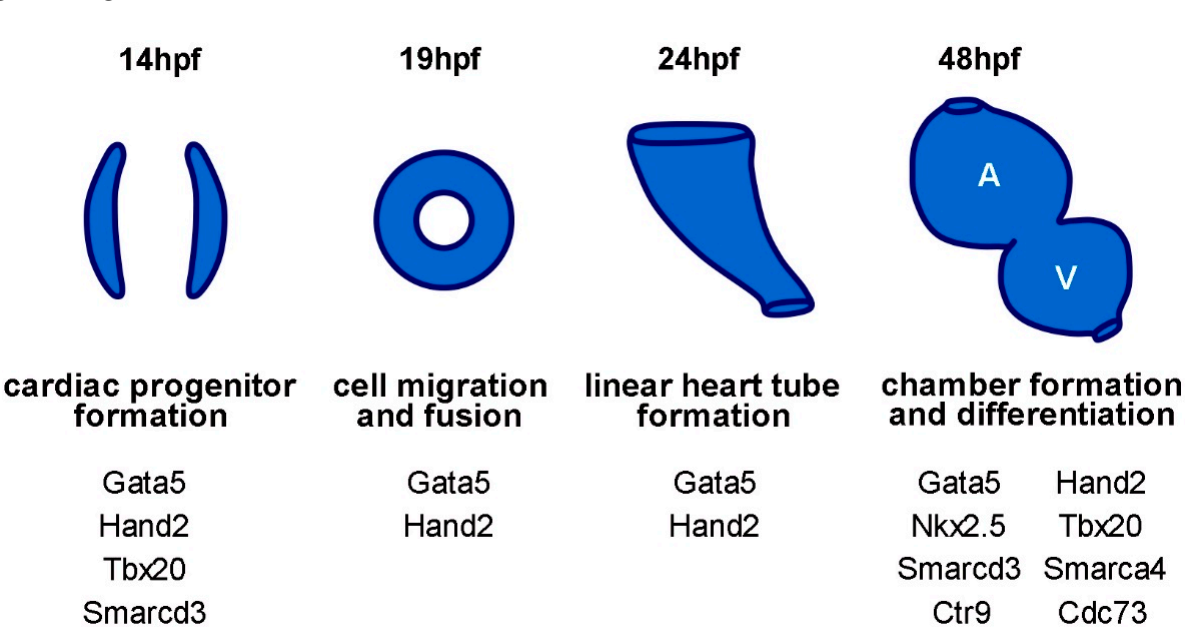
(Upper panel) Overview of zebrafish heart development. By 14 hpf, cardiac progenitors have emerged from the anterior lateral plate mesoderm. These cardiac precursors migrate and fuse at the midline to form a cone structure by 19 hpf. After one day of development, a beating linear heart tube has formed to propel circulation through the body. Cardiac chambers are clearly demarcated and looping has completed after two days of development. (Lower panel) Genes discussed in this review are listed below the developmental processes that they regulate. V, ventricle; A, atrium.

Over the last two decades the zebrafish has emerged as a powerful model organism for studying cardiac development. The mature zebrafish heart has a simplified structure, consisting of only one atrial and one ventricular chamber. Despite its anatomical differences with the four-chambered mammalian heart, the developmental mechanisms of zebrafish cardiogenesis closely parallel those of other vertebrate animal models (Figure 1). Cardiac progenitors in zebrafish are descended from cells located at the ventrolateral marginal region of the early blastula [7]. At the onset of gastrulation, these mesodermal cells are among the first to involute and migrate toward the embryonic axis. The activation of an evolutionarily conserved cardiac transcription program including GATA factors, Hand2, T-box proteins, and Nkx2.5 specifies cardiac progenitors within the anterior lateral plate mesoderm (ALPM), which subsequently migrate toward the midline of the embryo, turn on expression of cardiac sarcomere proteins, and fuse to form a beating heart tube. As has been shown in mammals, the continued growth and maturation of the zebrafish heart depend on the recruitment of additional cardiac progenitor cells from the second heart field (SHF), which make a large contribution to the distal ventricle and outflow tract [8].

Because of the transparency of zebrafish embryos and their amenability to embryonic and molecular manipulations, the simple zebrafish heart is a convenient vertebrate model for examining the specification of cardiac progenitors, heart tube formation, and the growth and maturation of the cardiac chambers. In this review, we will discuss recent discoveries made possible by the genetic and embryological tools available in the zebrafish model organism, with a focus on the novel functions of cardiac transcription factors and epigenetic and transcriptional regulatory proteins in the myocardium during cardiogenesis.

## 2. Cardiac Transcription Factors

### 2.1. GATA Factors Control Cardiomyocyte Progenitor Migration and Regulate Cardiac Fate Specification by Restricting Wnt Signaling

GATA factors function at the top of the hierarchy of cardiac genes, and regulate multiple aspects of cardiogenesis by both tissue autonomous and non-autonomous mechanisms. In zebrafish, *gata4*, *5*, and *6* are expressed in the ALPM and endoderm and control expression of myocardial and endodermal genes [9,10]. Similar to what has been observed in Gata4 knockout mice [11,12], loss of Gata5 function in zebrafish *faust* mutants disrupts migration of the endoderm to the midline [9,10]. Consistent with the crucial role the endoderm plays in migration of cardiac cells to the midline [11,13], *faust* mutants exhibit cardia bifida [9]. In addition to this non-tissue autonomous phenotype, *gata5*-deficient zebrafish embryos have a reduced number of cardiac progenitors, pointing to a cell intrinsic role for GATA factors in the production of cardiomyocytes [9]. Consistent with this view, overexpression of Gata5 expands the expression domains of cardiac genes including *nkx2.5* within the ALPM [9]. Additionally, zebrafish embryos lacking both Gata5 and Gata6 show a complete absence of *nkx2.5*-positive progenitor cells, highlighting a redundant requirement of GATA factors for cardiac fate specification [14].

Recent studies have also identified crosstalk between GATA factors and other critical cardiogenic signaling pathways [15,16,17]. For example, Tmem88a, a suppressor of canonical Wnt signaling, is downregulated in zebrafish *gata5/6* double morphants. Both excessive and reduced *tmem88a* expression disrupt *nkx2.5* expression in the ALPM, consistent with the biphasic function of Wnt signaling in cardiac development and highlighting a dosage-sensitive requirement for Tmem88a [16]. Similarly, knockdown of Tmem88a during directed cardiac differentiation of human ES cells increases Wnt/β-catenin signaling and results in a reduced number of cardiac progenitor cells [17], pointing to a critical role of Tmem88a in mediating crosstalk between GATA factors and the Wnt signaling pathway.

### 2.2. Hand2 Regulates Cardiac Differentiation and Morphogenesis

Hand2 is a transcription factor of the basic-helix-loop-helix family whose expression domain demarcates the heart forming region in the ALPM [18]. Forced expression of *hand2* in early zebrafish embryos promotes cardiac progenitor formation at the expense of the neighboring hematopoietic and endothelial cell lineages, suggesting an early impact of *hand2* on cardiac progenitor cell formation. Interestingly, the initial specification of cardiac progenitor cells does not require Hand2 activity as *nkx2.5*-positive cardiac progenitor cells do emerge from the ALPM in *hand2*-deficient embryos. However, *hand2* deficient cardiac progenitor cells are much less efficient at generating differentiated cardiomyocytes. As a result, *hand2*-deficient embryos have fewer cardiomyocytes and these cardiomyocytes are not sufficient to support endocardial differentiation [18,19].

Myocardial precursor form polarized epithelia in zebrafish embryos following their specification from the ALPM [20,21]. In *hand2* mutants, these polarized myocardial precursors fail to migrate and fuse at the midline, resulting in cardia bifida [22]. When transplanted into a wild-type host, *hand2* mutant cardiomyocytes are able to migrate to the midline and integrate into the heart tube, suggesting that the migration of cardiomyocytes is dependent on Hand2 function in their environment [23]. Fibronectin is a key component of the ECM surrounding the cardiac epithelium, and cardiac progenitor migration is sensitive to its expression levels [20]. Interestingly, Fibronectin accumulation is increased in *hand2* mutants and decreased upon overexpression of Hand2 [21,23]. Reduction of Fibronectin levels in *hand2* mutants restored migration of cardiac progenitors to the midline [23], indicating that Hand2 promotes the migration and fusion of the cardiac epithelium by limiting Fibronectin deposition and highlighting a mechanism by which a cardiac transcription factor prepares the embryonic environment for normal cardiac morphogenesis.

### 2.3. Nkx2.5 Maintains Cardiac Chamber Identity

Nkx2.5 is a homeodomain transcription factor that is one of the earliest markers of the cardiac lineage. Genetic studies in *Drosophila*, zebrafish, and mice have revealed divergent functions of Nkx2.5 during early heart development. In flies, Nkx2.5 activity is essential for cardiac specification, and *tinman/nkx2.5* mutants completely lack a dorsal vessel [24,25]. On the contrary, a heart tube forms normally in *nkx2.5*-deficient zebrafish and mouse embryos, but the subsequent differentiation and maturation of its chambers are impaired [26,27,28].

Loss-of-function of both Nkx2.5 and Nkx2.7, two zebrafish homologs of mammalian Nkx2.5 [29,30], results in a dysmorphic heart tube with an oversized, hyperplastic atrial chamber and a small ventricular chamber with a reduced number of cardiomyocytes [28,31]. In zebrafish, chamber-specific gene programs are established after two days of development such that distinct sets of genes are restricted to the atrium (*i.e.*, *amhc*) and the ventricle (*i.e.*, *vmhc*). Interestingly, *amhc*-positive cells are observed outside of the atrium near the arterial pole of the *nkx*-deficient hearts, suggesting that *nkx* genes are required for the maintenance of chamber identity [28,31]. Reintroduction of Nkx2.5 expression using a heat shock-inducible transgene during the time frame when cardiomyocytes are differentiating restores normal chamber-specific cardiomyocyte production in *nkx2.5* mutant embryos, suggesting that early expression of Nkx2.5 may prime cardiomyocytes for their later chamber identity [32]. Furthermore, the temporary overexpression of Nkx2.5 in *nkx2.5* mutants during embryonic stages permits normal cardiac function into adulthood, and demonstrates that in contrast to the established ongoing requirement of *nkx2.5* in mature cardiomyocytes of adult mice, Nkx2.5 is dispensable for adult zebrafish cardiac function [32]. Nevertheless, given the redundant roles of *nkx2.5* and *nkx2.7* during early cardiac development [28,31], more study is required to determine the full extent of *nkx* gene functions in the adult zebrafish heart.

### 2.4. Tbx20 Is Required for Cardiac Progenitor Formation

Tbx20 is an ancient T-box protein whose cardiac expression initiates at the time when cardiac progenitor cells emerge from mesoderm and persists to adulthood [33,34,35,36,37]. In mice, Tbx20 overexpression promotes cardiomyocyte proliferation whereas *Tbx20* deficiency results in impaired cardiomyocyte differentiation and cardiac developmental arrest after formation of the linear heart tube [38,39,40,41,42]. Ablation of Tbx20 activity in adult cardiomyocytes disrupts the structure of myofibrils and causes arrhythmia in both *Drosophila* and mouse models [36,43], and mutations in *tbx20* are associated with diverse cardiac pathologies including cardiomyopathy in humans [43,44,45,46,47,48,49].

While Tbx20’s function in the regulation of cardiac chamber morphogenesis and the maintenance of cardiac physiology and cellular structure appears to be conserved in all species examined, its role in cardiac progenitor cell formation is still unclear. In flies, the cardiac progenitor population is severely compromised when both *tbx20* homologues are downregulated [50], but *Tbx20* ablation in mice does not impact the formation of the cardiac crescent and the primitive heart tube forms in *Xenopus* and zebrafish *tbx20* morphants [39,40,41,42,51,52]. This has led to the conclusion that Tbx20 activity is dispensable during early cardiogenesis in vertebrates. However, our recent discovery that cardiac progenitor cells are diminished in zebrafish *tbx20* null mutant embryos argues against this hypothesis (Lu, Langenbacher and Chen, unpublished data). Further investigation is needed to determine whether compensatory mechanisms exist in the mouse to allow cardiac crescent formation in the absence of Tbx20 or if the zebrafish represents a transition state in the evolution of *tbx20* gene function in vertebrates.

## 3. Epigenetic and Transcriptional Regulators

The accessibility of regulatory DNA sequences to transcription factors and RNA polymerase II is dynamically regulated by covalent histone modifications, DNA methylation, and ATP-dependent chromatin remodeling. As a result, epigenetic regulation plays a critical role in driving cardiac gene expression and cardiac fate specification. Furthermore, epigenetic regulators control the morphogenesis and maturation of the heart. For example, loss of function of the Histone 3 Lysine 4 methyltransferases Smyd3 and Setd7 in zebrafish embryos produces severe defects in cardiac chamber morphogenesis, and Histone deacetylase activity controls valvulogenesis in zebrafish by promoting myocardial expression of Bmp4 at the atrioventricular boundary [53,54]. Here we will focus on two complexes, BAF and PAF1, which play a central role in transcriptional regulation of zebrafish cardiac development.

### 3.1. BAF Chromatin Remodeling Complex Induces a Cardiac Mesoderm Fate

The SWI/SNF (switching defective/sucrose non-fermenting) complex is an ATP-dependent chromatin remodeling complex that was initially purified from yeast. Its vertebrate homolog, the BAF (Brg1/Brm-associated factor) complex, contains 12 protein subunits including Baf60c/Smarcd3 and Brg1/Smarca4. Many components of the BAF complex are significantly upregulated in patients with hypertrophic cardiomyopathy [55,56,57] and are indirectly implicated in the pathogenesis of CHD [58,59]. The BAF complex regulates cardiogenesis by interacting with cardiac transcription factors like Tbx1, Tbx5, Nkx2.5, and Gata4 [60,61,62]. Loss of function of Brg1/Smarca4 and Baf60c/Smarcd3 in mouse embryos results in a wide range of congenital heart defects and the cardiac defects caused by *Brg1/Smarca4* haploinsufficiency are exacerbated in an *Nkx2.5*, *Tbx20*, or *Tbx5* heterozygous background, highlighting the genetic interaction between the BAF complex and the central members of the cardiac transcription program [55,60,63].

In fish, loss of function of Smarca4 or Smarcd3 causes progressive morphological and functional deterioration of the heart [61,63,64]. In addition, simultaneous overexpression of Gata5 and Smarcd3 in zebrafish embryos leads to expanded expression of cardiac genes and an enlarged heart [61]. Similarly, Gata4/Smarcd3/Tbx5 expression promotes cardiomyocyte differentiation from non-cardiogenic mesoderm in mice [65], suggesting that GATA factors and the BAF complex have a conserved and joint role in promoting the cardiac gene program. Interestingly, when cells overexpressing both Gata5 and Smarcd3 were transplanted to regions outside of the cardiac field of wild-type host zebrafish embryos, they often contributed to the cardiac region of the ALPM and differentiated into cardiomyocytes, endocardium, and smooth muscle [61]. These findings from the zebrafish model suggest that Gata5 and Smarcd3 can induce a cardiac fate in a cell regardless of its position within the embryo as well as promote the differentiation of multiple cardiac lineages.

### 3.2. The PAF1 Complex Is Involved in Cardiac Progenitor Formation and Cardiomyocyte Differentiation

The Polymerase Associated Factor 1 complex (PAF1C) is an evolutionarily conserved protein complex that accompanies RNA PolII through the entire open reading frame of transcribed genes and regulates multiple transcriptional processes, including initiation, elongation, termination, 3′ end formation, and transcription-coupled histone modification (for review see [66]). The PAF1C consists of five components: Leo1, Ctr9, Cdc73, Paf1, and Rtf1. Genetic studies have suggested a requirement of PAF1C components in multiple biological processes including cell cycle regulation, somite and neural crest development, and maintenance of stem cell pluripotency [67,68,69,70]. Loss of any one of the PAF1C components results in abnormal heart development in zebrafish, but, interestingly, the individual proteins of the PAF1C play distinct roles during cardiogenesis. Leo1 is required for chamber morphogenesis and the differentiation of cardiomyocytes at the atrioventricular boundary of the heart, while Ctr9, Cdc73, and Paf1 are required for generating an appropriate number of cardiomyocytes and elongation of the heart tube [69,71]. Strikingly, loss of Rtf1 causes the most severe cardiac phenotype; ablation of Rtf1 activity eliminates the cardiac progenitor population and abolishes heart formation [71]. Upon transplantation, *rtf1*-deficient donor cells do not contribute to the heart in wild type embryos, while wild-type donor cells are able to form cardiomyocytes in *rtf1*-deficient embryos, indicating that Rtf1 regulates cardiac development in a cell autonomous manner [71]. It is currently not known how individual components of the PAF1C assume different roles in cardiogenesis or why Rtf1 deficiency causes such a dramatic effect on heart formation. Given the multifunctional role that the PAF1C plays in transcription regulation, one possible explanation is that PAF1C members differentially regulate a transcriptional event critical for cardiogenesis. Alternatively, it is intriguing to note that Rtf1 is loosely associated with the PAF1C in metazoans and that PAF1C-independent regulation of transcriptional elongation by Rtf1 has been observed in fission yeast and HeLa cells [71,72,73,74,75,76,77]. Continued study is essential to clarify the biological significance of individual PAF1C components and provide insights into their regulation of the cardiac transcription program.

## 4. Summary

A multitude of novel findings in zebrafish have helped to identify the critical mechanisms by which cardiac transcription factors, chromatin remodeling proteins, and general transcriptional regulators shape the developing heart and demonstrate the power of using zebrafish as a model to increase our knowledge about cardiogenesis. A deeper understanding of the steps of cardiogenesis is critical for deciphering the molecular mechanisms underlying congenital heart defects and developing novel regenerative therapies for cardiovascular disease.

Unlike mammalian hearts, the zebrafish heart shows robust regenerative capabilities and can restore a fully functional heart even after severe injury. Following ventricular resection, new cardiomyocytes regenerate via the de-differentiation and proliferation of existing cardiomyocytes [78], and recent studies highlight the reactivation and function of embryonic cardiac gene programs during regeneration. Cardiomyocytes throughout the subepicardial ventricular layer trigger expression of *gata4* before beginning to proliferate and contribute to heart regeneration [79,80]. Increased expression of *hand2*, *nkx2.5*, *tbx5*, and *tbx20* has been observed in the myocardium surrounding sites of injury, and higher levels of *gata5* and *hand2* expression were also detected within the injury site endocardium [81,82,83]. Strikingly, overexpression of Hand2 in adult zebrafish cardiomyocytes augments their proliferative and regenerative capacity [81]. Future studies exploring the functions of cardiac transcription factors, chromatin remodeling proteins, and general transcriptional regulators after injury may also lead to new therapies for cardiac regeneration and repair.

## References

[1] Bruneau B.G. (2013). Signaling and transcriptional networks in heart development and regeneration. Cold Spring Harb. Perspect. Biol..

[2] Paige S.L., Plonowska K., Xu A., Wu S.M. (2015). Molecular regulation of cardiomyocyte differentiation. Circ. Res..

[3] Rana M.S., Christoffels V.M., Moorman A.F. (2013). A molecular and genetic outline of cardiac morphogenesis. Acta Physiol. (Oxford, England).

[4] Chang C.P., Bruneau B.G. (2012). Epigenetics and cardiovascular development. Annu. Rev. Physiol..

[5] Boland M.J., Nazor K.L., Loring J.F. (2014). Epigenetic regulation of pluripotency and differentiation. Circ. Res..

[6] Nuhrenberg T., Gilsbach R., Preissl S., Schnick T., Hein L. (2014). Epigenetics in cardiac development, function, and disease. Cell Tissue Res..

[7] Stainier D.Y., Lee R.K., Fishman M.C. (1993). Cardiovascular development in the zebrafish. I. Myocardial fate map and heart tube formation. Development.

[8] Zhou Y., Cashman T.J., Nevis K.R., Obregon P., Carney S.A., Liu Y., Gu A., Mosimann C., Sondalle S., Peterson R.E. (2011). Latent TGF-β binding protein 3 identifies a second heart field in zebrafish. Nature.

[9] Reiter J.F., Alexander J., Rodaway A., Yelon D., Patient R., Holder N., Stainier D.Y. (1999). Gata5 is required for the development of the heart and endoderm in zebrafish. Genes Dev..

[10] Reiter J.F., Kikuchi Y., Stainier D.Y. (2001). Multiple roles for Gata5 in zebrafish endoderm formation. Development.

[11] Kuo C.T., Morrisey E.E., Anandappa R., Sigrist K., Lu M.M., Parmacek M.S., Soudais C., Leiden J.M. (1997). Gata4 transcription factor is required for ventral morphogenesis and heart tube formation. Genes Dev..

[12] Molkentin J.D., Lin Q., Duncan S.A., Olson E.N. (1997). Requirement of the transcription factor Gata4 for heart tube formation and ventral morphogenesis. Genes Dev..

[13] Narita N., Bielinska M., Wilson D.B. (1997). Wild-type endoderm abrogates the ventral developmental defects associated with GATA-4 deficiency in the mouse. Dev. Biol..

[14] Holtzinger A., Evans T. (2007). Gata5 and Gata6 are functionally redundant in zebrafish for specification of cardiomyocytes. Dev. Biol..

[15] Moskowitz I.P., Wang J., Peterson M.A., Pu W.T., Mackinnon A.C., Oxburgh L., Chu G.C., Sarkar M., Berul C., Smoot L. (2011). Transcription factor genes Smad4 and Gata4 cooperatively regulate cardiac valve development. [Corrections]. Proc. Nal. Acad. Sci. USA.

[16] Novikov N., Evans T. (2013). Tmem88a mediates GATA-dependent specification of cardiomyocyte progenitors by restricting wnt signaling. Development.

[17] Palpant N.J., Pabon L., Rabinowitz J.S., Hadland B.K., Stoick-Cooper C.L., Paige S.L., Bernstein I.D., Moon R.T., Murry C.E. (2013). Transmembrane protein 88: A wnt regulatory protein that specifies cardiomyocyte development. Development.

[18] Schoenebeck J.J., Keegan B.R., Yelon D. (2007). Vessel and blood specification override cardiac potential in anterior mesoderm. Dev. Cell.

[19] Palencia-Desai S., Rost M.S., Schumacher J.A., Ton Q.V., Craig M.P., Baltrunaite K., Koenig A.L., Wang J., Poss K.D., Chi N.C. (2015). Myocardium and BMP signaling are required for endocardial differentiation. Development.

[20] Trinh L.A., Stainier D.Y. (2004). Fibronectin regulates epithelial organization during myocardial migration in zebrafish. Dev. cell.

[21] Trinh L.A., Yelon D., Stainier D.Y. (2005). Hand2 regulates epithelial formation during myocardial diferentiation. Curr. Biol.: CB.

[22] Yelon D., Ticho B., Halpern M.E., Ruvinsky I., Ho R.K., Silver L.M., Stainier D.Y. (2000). The BHLH transcription factor hand2 plays parallel roles in zebrafish heart and pectoral fin development. Development.

[23] Garavito-Aguilar Z.V., Riley H.E., Yelon D. (2010). Hand2 ensures an appropriate environment for cardiac fusion by limiting fibronectin function. Development.

[24] Azpiazu N., Frasch M. (1993). Tinman and bagpipe: Two homeo box genes that determine cell fates in the dorsal mesoderm of drosophila. Genes Dev..

[25] Bodmer R. (1993). The gene tinman is required for specification of the heart and visceral muscles in drosophila. Development.

[26] Lyons I., Parsons L.M., Hartley L., Li R., Andrews J.E., Robb L., Harvey R.P. (1995). Myogenic and morphogenetic defects in the heart tubes of murine embryos lacking the homeo box gene Nkx2–5. Genes Dev..

[27] Tanaka M., Wechsler S.B., Lee I.W., Yamasaki N., Lawitts J.A., Izumo S. (1999). Complex modular *cis*-acting elements regulate expression of the cardiac specifying homeobox gene Csx/Nkx2.5. Development.

[28] Targoff K.L., Schell T., Yelon D. (2008). Nkx genes regulate heart tube extension and exert differential effects on ventricular and atrial cell number. Dev. Biol..

[29] Chen J.N., Fishman M.C. (1996). Zebrafish tinman homolog demarcates the heart field and initiates myocardial differentiation. Development.

[30] Lee K.H., Xu Q., Breitbart R.E. (1996). A new tinman-related gene, Nkx2.7, anticipates the expression of Nkx2.5 and Nkx2.3 in zebrafish heart and pharyngeal endoderm. Dev. Biol..

[31] Targoff K.L., Colombo S., George V., Schell T., Kim S.H., Solnica-Krezel L., Yelon D. (2013). Nkx genes are essential for maintenance of ventricular identity. Development.

[32] George V., Colombo S., Targoff K.L. (2015). An early requirement for Nkx2.5 ensures the first and second heart field ventricular identity and cardiac function into adulthood. Dev. Biol..

[33] Ahn D.G., Ruvinsky I., Oates A.C., Silver L.M., Ho R.K. (2000). TBX20, a new vertebrate T-box gene expressed in the cranial motor neurons and developing cardiovascular structures in zebrafish. Mech. Dev..

[34] Griffin K.J., Stoller J., Gibson M., Chen S., Yelon D., Stainier D.Y., Kimelman D. (2000). A conserved role for H15-related T-box transcription factors in zebrafish and drosophila heart formation. Dev. Biol..

[35] Brown D.D., Binder O., Pagratis M., Parr B.A., Conlon F.L. (2003). Developmental expression of the Xenopus laevis TBX20 orthologue. Dev. Genes Evol..

[36] Shen T., Aneas I., Sakabe N., Dirschinger R.J., Wang G., Smemo S., Westlund J.M., Cheng H., Dalton N., Gu Y. (2011). TBX20 regulates a genetic program essential to adult mouse cardiomyocyte function. J. Clin. Investig..

[37] Kraus F., Haenig B., Kispert A. (2001). Cloning and expression analysis of the mouse T-box gene TBX20. Mech. Dev..

[38] Chakraborty S., Yutzey K.E. (2012). TBX20 regulation of cardiac cell proliferation and lineage specialization during embryonic and fetal development *in vivo*. Dev. Biol..

[39] Cai C.L., Zhou W., Yang L., Bu L., Qyang Y., Zhang X., Li X., Rosenfeld M.G., Chen J., Evans S. (2005). T-box genes coordinate regional rates of proliferation and regional specification during cardiogenesis. Development.

[40] Singh M.K., Christoffels V.M., Dias J.M., Trowe M.O., Petry M., Schuster-Gossler K., Burger A., Ericson J., Kispert A. (2005). TBX20 is essential for cardiac chamber differentiation and repression of TBX2. Development.

[41] Stennard F.A., Costa M.W., Lai D., Biben C., Furtado M.B., Solloway M.J., McCulley D.J., Leimena C., Preis J.I., Dunwoodie S.L. (2005). Murine T-box transcription factor TBX20 acts as a repressor during heart development, and is essential for adult heart integrity, function and adaptation. Development.

[42] Takeuchi J.K., Mileikovskaia M., Koshiba-Takeuchi K., Heidt A.B., Mori A.D., Arruda E.P., Gertsenstein M., Georges R., Davidson L., Mo R. (2005). TBX20 dose-dependently regulates transcription factor networks required for mouse heart and motoneuron development. Development.

[43] Qian L., Mohapatra B., Akasaka T., Liu J., Ocorr K., Towbin J.A., Bodmer R. (2008). Transcription factor neuromancer/TBX20 is required for cardiac function in drosophila with implications for human heart disease. Proc. Natl. Acad. Sci. USA.

[44] Kirk E.P., Sunde M., Costa M.W., Rankin S.A., Wolstein O., Castro M.L., Butler T.L., Hyun C., Guo G., Otway R. (2007). Mutations in cardiac t-box factor gene TBX20 are associated with diverse cardiac pathologies, including defects of septation and valvulogenesis and cardiomyopathy. Am. J. Hum. Genet..

[45] Liu C., Shen A., Li X., Jiao W., Zhang X., Li Z. (2008). T-box transcription factor TBX20 mutations in Chinese patients with congenital heart disease. Eur. J. Med. Genet..

[46] Hammer S., Toenjes M., Lange M., Fischer J.J., Dunkel I., Mebus S., Grimm C.H., Hetzer R., Berger F., Sperling S. (2008). Characterization of TBX20 in human hearts and its regulation by TFAP2. J. Cell. Biochem..

[47] Posch M.G., Gramlich M., Sunde M., Schmitt K.R., Lee S.H., Richter S., Kersten A., Perrot A., Panek A.N., Al Khatib I.H. (2010). A gain-of-function TBX20 mutation causes congenital atrial septal defects, patent foramen ovale and cardiac valve defects. J. Med. Genet..

[48] De Jong R.N., Truffault V., Diercks T., Ab E., Daniels M.A., Kaptein R., Folkers G.E. (2008). Structure and DNA binding of the human Rtf1 Plus3 domain. Structure.

[49] Pan Y., Geng R., Zhou N., Zheng G.F., Zhao H., Wang J., Zhao C.M., Qiu X.B., Yang Y.Q., Liu X.Y. (2015). TBX20 loss-of-function mutation contributes to double outlet right ventricle. Int. J. Mol. Med..

[50] Qian L., Liu J., Bodmer R. (2005). Neuromancer TBX20-related genes (H15/midline) promote cell fate specification and morphogenesis of the drosophila heart. Dev. Biol..

[51] Szeto D.P., Griffin K.J., Kimelman D. (2002). HrT is required for cardiovascular development in zebrafish. Development.

[52] Brown D.D., Martz S.N., Binder O., Goetz S.C., Price B.M., Smith J.C., Conlon F.L. (2005). TBX5 and TBX20 act synergistically to control vertebrate heart morphogenesis. Development.

[53] Kim J.D., Kim E., Koun S., Ham H.J., Rhee M., Kim M.J., Huh T.L. (2015). Proper activity of histone H3 lysine 4 (H3K4) methyltransferase is required for morphogenesis during zebrafish cardiogenesis. Mol. Cells.

[54] Kim Y.S., Kim M.J., Koo T.H., Kim J.D., Koun S., Ham H.J., Lee Y.M., Rhee M., Yeo S.Y., Huh T.L. (2012). Histone deacetylase is required for the activation of Wnt/β-catenin signaling crucial for heart valve formation in zebrafish embryos. Biochem. Biophys. Res. Commun..

[55] Hang C.T., Yang J., Han P., Cheng H.-L., Shang C., Ashley E., Zhou B., Chang C.-P. (2010). Chromatin regulation by Brg1 underlies heart muscle development and disease. Nature.

[56] Cui H., Schlesinger J., Schoenhals S., Tonjes M., Dunkel I., Meierhofer D., Cano E., Schulz K., Berger M.F., Haack T. (2015). Phosphorylation of the chromatin remodeling factor DPF3a induces cardiac hypertrophy through releasing hey repressors from DNA. Nucl. Acids Res..

[57] Lange M., Kaynak B., Forster U.B., Tonjes M., Fischer J.J., Grimm C., Schlesinger J., Just S., Dunkel I., Krueger T. (2008). Regulation of muscle development by DPF3, a novel histone acetylation and methylation reader of the baf chromatin remodeling complex. Genes Dev..

[58] Bajpai R., Chen D.A., Rada-Iglesias A., Zhang J., Xiong Y., Helms J., Chang C.P., Zhao Y., Swigut T., Wysocka J. (2010). CHD7 cooperates with PBAF to control multipotent neural crest formation. Nature.

[59] Kitagawa H., Fujiki R., Yoshimura K., Mezaki Y., Uematsu Y., Matsui D., Ogawa S., Unno K., Okubo M., Tokita A. (2003). The chromatin-remodeling complex WINAC targets a nuclear receptor to promoters and is impaired in Williams syndrome. Cell.

[60] Lickert H., Takeuchi J.K., Von Both I., Walls J.R., McAuliffe F., Adamson S.L., Henkelman R.M., Wrana J.L., Rossant J., Bruneau B.G. (2004). Baf60c is essential for function of BAF chromatin remodelling complexes in heart development. Nature.

[61] Lou X., Deshwar A.R., Crump J.G., Scott I.C. (2011). Smarcd3b and Gata5 promote a cardiac progenitor fate in the zebrafish embryo. Development.

[62] Chen L., Fulcoli F.G., Ferrentino R., Martucciello S., Illingworth E.A., Baldini A. (2012). Transcriptional control in cardiac progenitors: TBX1 interacts with the BAF chromatin remodeling complex and regulates wnt5a. PLoS Genet..

[63] Takeuchi J.K., Lou X., Alexander J.M., Sugizaki H., Delgado-Olguin P., Holloway A.K., Mori A.D., Wylie J.N., Munson C., Zhu Y. (2011). Chromatin remodelling complex dosage modulates transcription factor function in heart development. Nat. Commun..

[64] Gregg R.G., Willer G.B., Fadool J.M., Dowling J.E., Link B.A. (2003). Positional cloning of the young mutation identifies an essential role for the brahma chromatin remodeling complex in mediating retinal cell differentiation. Proc. Natl. Acad. Sci. USA.

[65] Takeuchi J.K., Bruneau B.G. (2009). Directed transdifferentiation of mouse mesoderm to heart tissue by defined factors. Nature.

[66] Tomson B.N., Arndt K.M. (2013). The many roles of the conserved eukaryotic PAF1 complex in regulating transcription, histone modifications, and disease states. Biochim. Biophys. Acta (BBA)—Gene Regul. Mech..

[67] Koch C., Wollmann P., Dahl M., Lottspeich F. (1999). A role for Ctr9p and PAF1p in the regulation G1 cyclin expression in yeast. Nucl. Acids Res..

[68] Akanuma T., Koshida S., Kawamura A., Kishimoto Y., Takada S. (2007). PAF1 complex homologues are required for notch-regulated transcription during somite segmentation. EMBO Rep..

[69] Nguyen C.T., Langenbacher A., Hsieh M., Chen J.N. (2010). The PAF1 complex component Leo1 is essential for cardiac and neural crest development in zebrafish. Dev. Biol..

[70] Ding L., Paszkowski-Rogacz M., Nitzsche A., Slabicki M.M., Heninger A.K., de Vries I., Kittler R., Junqueira M., Shevchenko A., Schulz H. (2009). A genome-scale RNAi screen for Oct4 modulators defines a role of the PAF1 complex for embryonic stem cell identity. Cell Stem Cell.

[71] Langenbacher A.D., Nguyen C.T., Cavanaugh A.M., Huang J., Lu F., Chen J.N. (2011). The PAF1 complex differentially regulates cardiomyocyte specification. Dev. Biol..

[72] Mbogning J., Nagy S., Page V., Schwer B., Shuman S., Fisher R.P., Tanny J.C. (2013). The PAF complex and Prf1/Rtf1 delineate distinct Cdk9-dependent pathways regulating transcription elongation in fission yeast. PLoS Genet..

[73] Cao Q.F., Yamamoto J., Isobe T., Tateno S., Murase Y., Chen Y., Handa H., Yamaguchi Y. (2015). Characterization of the human transcription elongation factor Rtf1: Evidence for nonoverlapping functions of Rtf1 and the PAF1 complex. Mol. Cell. Biol..

[74] Rozenblatt-Rosen O., Hughes C.M., Nannepaga S.J., Shanmugam K.S., Copeland T.D., Guszczynski T., Resau J.H., Meyerson M. (2005). The parafibromin tumor suppressor protein is part of a human PAF1 complex. Mol. Cell. Biol..

[75] Zhu B., Mandal S.S., Pham A.D., Zheng Y., Erdjument-Bromage H., Batra S.K., Tempst P., Reinberg D. (2005). The human PAF complex coordinates transcription with events downstream of RNA synthesis. Genes Dev..

[76] Adelman K., Wei W., Ardehali M.B., Werner J., Zhu B., Reinberg D., Lis J.T. (2006). Drosophila PAF1 modulates chromatin structure at actively transcribed genes. Mol. Cell. Biol..

[77] Nordick K., Hoffman M.G., Betz J.L., Jaehning J.A. (2008). Direct interactions between the PAF1 complex and a cleavage and polyadenylation factor are revealed by dissociation of PAF1 from RNA polymerase ii. Eukaryot. Cell.

[78] Jopling C., Sleep E., Raya M., Marti M., Raya A., Belmonte J.C.I. (2010). Zebrafish heart regeneration occurs by cardiomyocyte dedifferentiation and proliferation. Nature.

[79] Kikuchi K., Holdway J.E., Werdich A.A., Anderson R.M., Fang Y., Egnaczyk G.F., Evans T., MacRae C.A., Stainier D.Y.R., Poss K.D. (2010). Primary contribution to zebrafish heart regeneration by Gata4+ cardiomyocytes. Nature.

[80] Gupta V., Gemberling M., Karra R., Rosenfeld G.E., Evans T., Poss K.D. (2013). An injury-responsive Gata4 program shapes the zebrafish cardiac ventricle. Curr. Biol..

[81] Schindler Y.L., Garske K.M., Wang J., Firulli B.A., Firulli A.B., Poss K.D., Yelon D. (2014). Hand2 elevates cardiomyocyte production during zebrafish heart development and regeneration. Development.

[82] Lepilina A., Coon A.N., Kikuchi K., Holdway J.E., Roberts R.W., Burns C.G., Poss K.D. (2006). A dynamic epicardial injury response supports progenitor cell activity during zebrafish heart regeneration. Cell.

[83] Kikuchi K., Holdway J.E., Major R.J., Blum N., Dahn R.D., Begemann G., Poss K.D. (2011). Retinoic acid production by endocardium and epicardium is an injury response essential for zebrafish heart regeneration. Dev. Cell.

